# PB.04: MRI In lobular and mixed lobular/ductal carcinomas: can we preselect cases based on imaging appearance?

**DOI:** 10.1186/bcr3506

**Published:** 2013-11-08

**Authors:** MM Hoosein, L Grosvenor, D Lister, M Al-Attar

**Affiliations:** 1 University Hospitals of Leicester, UK

## Introduction

Lobular carcinoma presents a diagnostic challenge. Imaging and clinical findings are usually subtle. In the current climate it imposes extra strain on our resources. Our aim was to assess contribution of MRI in the preoperative local staging of lobular and mixed lobular/ductal carcinoma and to evaluate whether we can select cases for preoperative MRI based on mammographic appearance.

## Methods

A retrospective review of data provided by the local breast cancer database was performed. Patients with confirmed lobular or mixed lobular/ductal carcinomas that had MRI staging during a 5-year period were identified. Imaging and histopathology reports were reviewed. A total of 381 cancers were diagnosed in the study period. Ninety-one patients had breast MRI. Four cases were excluded as no final histology was available. Eighty-seven patients (mean age 58) with 89 involved breasts constituted our study population.

## Results

Breakdown of mammographic lesions was as follows: PD, 39 (43.8%); masses, 20 (22.4%); ASD, 18 (20.2%); lymphoedema, one (1.1%); calcifications, two (2.2%); occult/subtle, nine (10.1%). MRI had a positive contribution in 27/83 cases (32.5%), did not add any further information in 49/83 cases (59%) and a negative contribution in only 7/83 cases (8.4%). MRI notably identified greater disease extent, multifocal and contralateral disease. MRI was most useful in assessing disease extent when lobular carcinoma presented as PD or mammographically occult and was of least benefit when presenting as a mass lesion.

## Conclusion

MRI had no advantage over conventional imaging in the majority of lobular and mixed lobular/ductal cancers presenting as a focal mass lesion. Its application could be tailored more specifically to assess nonmass lesions.

